# PRRSV N protein antagonizes host antiviral immune responses by upregulating HSPA1B to induce autophagic degradation of Fos-related antigen 1

**DOI:** 10.1128/mbio.01091-26

**Published:** 2026-08-21

**Authors:** Xiao Liu, Jianwu Zhang, Xiaoyang Yan, Yinan Meng, Haozhe Wang, Fang Lv, Bo Peng, Zifang Zheng, Yang Li, Yingtong Feng, Lele Xu, Shuqi Xiao

**Affiliations:** 1 State Key Laboratory of Animal Disease Control and Prevention, College of Veterinary Medicine, Lanzhou University, Lanzhou Veterinary Research Institute, Chinese Academy of Agricultural Sciences 111658, Lanzhou, Gansu, China; 2 College of Veterinary Medicine, Northwest A&F University 12469, Yangling, Shaanxi, China; Dana-Farber Cancer Institute, Boston, Massachusetts, USA

**Keywords:** PRRSV, CSF2, FRA1, HSPA1B, chaperone-mediated autophagy

## Abstract

Porcine reproductive and respiratory syndrome virus (PRRSV) infection often causes severe immunosuppression in pigs. However, the mechanisms by which PRRSV antagonizes host antiviral immune responses remain incompletely understood. We found that granulocyte-macrophage colony-stimulating factor (CSF2) was significantly upregulated in porcine alveolar macrophages (PAMs) upon PRRSV infection. CSF2 upregulation inhibits PRRSV replication by promoting IL15 expression, and Fos-related antigen 1 (FRA1) enhances the antiviral activity of the CSF2-IL15 axis via transcriptional regulation. Further investigation revealed that the PRRSV N protein directly interacts with HSPA1B and activates HSPA1B-mediated chaperone-mediated autophagy (CMA) to degrade FRA1, thereby antagonizing host antiviral immunity. Our study reveals that the PRRSV N protein promotes FRA1 autophagic degradation by upregulating heat shock protein HSPA1B, thereby suppressing the host CSF2-IL15 antiviral pathway. This study provides new insights into the arms race between PRRSV and the host, as well as novel perspectives for the development of anti-PRRSV infection strategies.

**IMPORTANCE:**

Focusing on the core scientific issue of PRRSV immune evasion, this study identifies a novel pathway by which host FRA1 restricts viral infection through regulating the CSF2-IL15 cytokine axis. It reveals that the PRRSV N protein hijacks HSPA1B-mediated chaperone-mediated autophagy to target and degrade the transcription factor FRA1, thereby dismantling the host CSF2-IL15 antiviral defense and advancing the understanding of PRRSV-related immunosuppression. These findings elucidate a new paradigm in the host-pathogen tug-of-war, whereby viruses exploit autophagy machinery to eliminate critical immune regulators. This work also provides mechanistic insights for the development of intervention strategies targeting CMA and cytokine homeostasis.

## INTRODUCTION

PRRSV is a significant pathogen threatening the global swine industry, and its infection is often accompanied by severe immunosuppression and secondary infections (1, 2). Recent studies indicate that PRRSV evades host immune surveillance through multiple mechanisms and suppresses the cellular antiviral immune response, particularly by inhibiting the interferon signaling pathway, which is a core strategy for its immune evasion and persistent infection (3, 4). Additionally, PRRSV further weakens the host’s immune defense network by regulating cellular apoptosis, autophagy, and inflammatory cytokine pathways (5–7). Thus, elucidating the specific molecular mechanisms by which macrophages regulate cytokines for antiviral defense and how PRRSV antagonizes this mechanism to facilitate its own infection are key issues in the prevention and control of PRRSV.

In the complex defense network of the host against viral infection, cytokines, as central mediators of immune regulation, play an irreplaceable role by coordinating innate and adaptive immune responses (8, 9). When viruses breach physical barriers and invade the host, pattern recognition receptors rapidly recognize viral nucleic acids or protein components, triggering a cytokine cascade dominated by type I and type III interferons (IFNs) (10, 11). IFNs activate the JAK-STAT signaling pathway, inducing the expression of hundreds of interferon-stimulated genes (ISGs) to directly suppress viral replication (12, 13). Concurrently, macrophages secrete pro-inflammatory cytokines, amplifying local inflammatory responses to restrict viral replication. Furthermore, chemokines combat viral infection by recruiting NK cells, neutrophils, and T cells to the site of infection (14, 15). However, due to the powerful ability of PRRSV to suppress the host immune response (16), analyzing the antiviral defense response of macrophages is an urgent problem to be solved.

The immune evasion capabilities of PRRSV are closely linked to its precise disruption of the host’s antiviral defense system. Recent studies have shown that PRRSV not only inhibits the activation of the host’s innate immune signaling pathways but also hijacks and exploits host protein degradation machinery to dismantle core antiviral proteins, thereby creating a favorable environment of "immune-privileged" for viral replication (17–21). For instance, PRRSV GP5 inhibits the CMV-mediated innate immune pathway by targeting LAMP2A, thereby promoting its own replication (22). PRRSV Nsp2 promotes the autophagic degradation of adaptor protein SH3KBP1 to antagonize host innate immune responses by enhancing K63-linked polyubiquitination of RIG-I (4). Notably, the virus even hijacks the host autophagy-lysosome system to induce lysosome-dependent degradation of critical antiviral proteins, further weakening cell-autonomous antiviral defenses (6). The synergistic effect of these strategies enables PRRSV to achieve efficient replication and continuous infection within the host cells. Thus, deciphering the antiviral defense mechanisms of macrophages and PRRSV’s counteraction strategies not only enriches the host’s antiviral strategies but also yields novel insights into the arms race between PRRSV and the host.

## RESULTS

### CSF2 inhibits PRRSV replication

**Fig 1:**
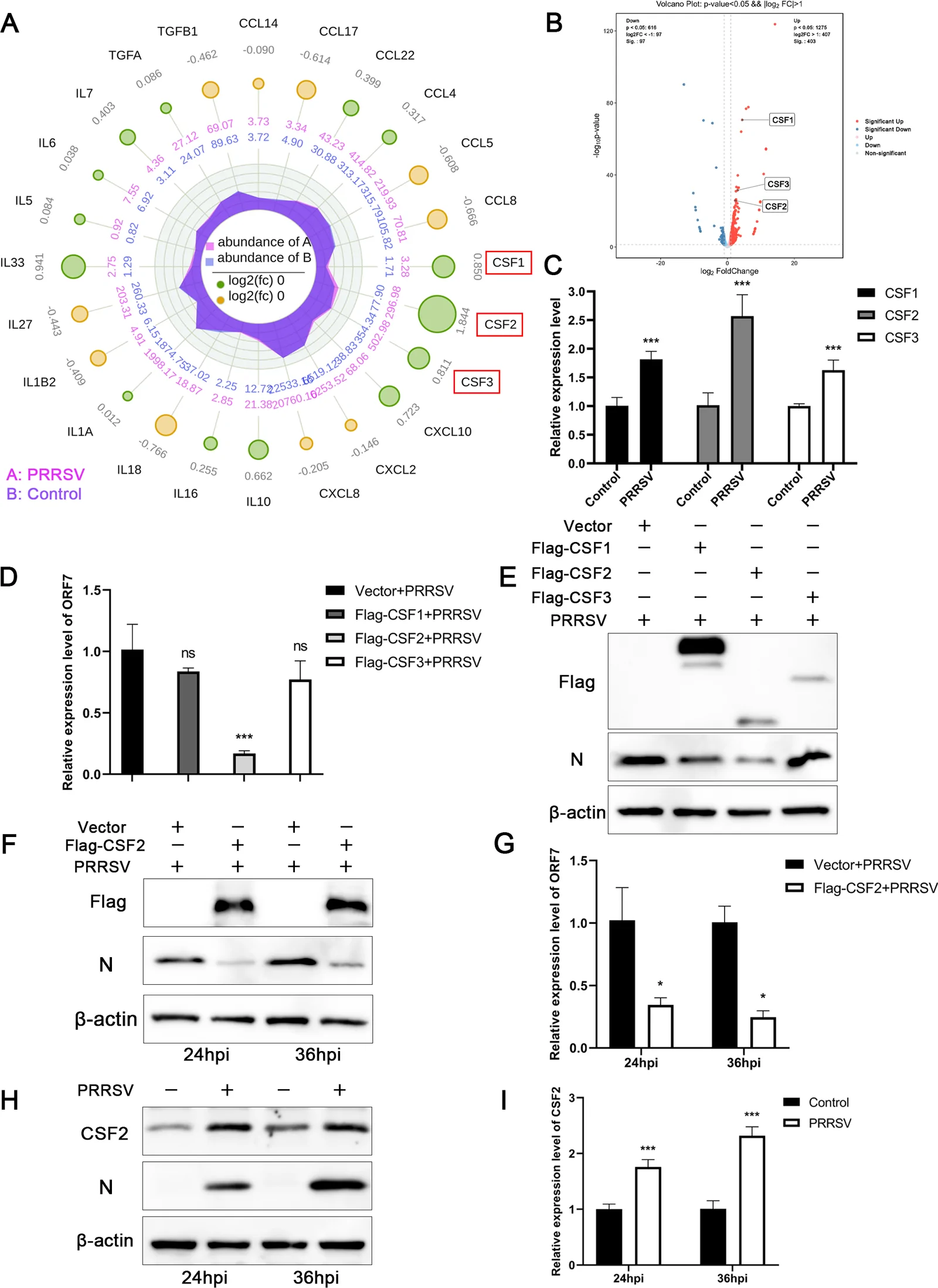
CSF2 inhibits PRRSV replication. (**A and B**) PAMs were infected with PRRSV, followed by transcriptomic analysis. (**C**) MARC-145 cells were infected with PRRSV, and the mRNA expression levels of CSF1, CSF2, and CSF3 were detected at 24 hpi. (**D and E**) MARC-145 cells were transfected with overexpression plasmids for CSF1, CSF2, or CSF3, then infected with PRRSV, and the expression levels of the PRRSV N protein and ORF7 gene were detected at 24 hpi. (**F and G**) MARC-145 cells were transfected with the CSF2 overexpression plasmid, then infected with PRRSV, and the expression levels of the PRRSV N protein and ORF7 gene were detected at 24 and 36 hpi. (**H and I**) MARC-145 cells were infected with PRRSV, and the protein and mRNA expression levels of CSF2 were detected at 24 and 36 hpi. *, *P*  <  0.05; **, *P*  <  0.01; ***, *P*  <  0.001 compared with the corresponding control.

Alveolar macrophages play pivotal roles in resisting respiratory viral infections (23, 24). However, the antiviral defense responses mounted by porcine alveolar macrophages (PAMs) upon sensing viral infection remain incompletely understood. To investigate the changes in cytokine expression in PAMs after PRRSV infection, we performed transcriptome sequencing analysis on PRRSV-infected PAMs. The results showed that PRRSV infection upregulated the expression of multiple cytokines, among which the colony-stimulating factor family genes (CSF1, CSF2, and CSF3) were all significantly upregulated (Fig. 1A and B). To further verify the effect of PRRSV infection on the expression of CSF1, CSF2, and CSF3, we infected MARC-145 cells with PRRSV GD-HD (MOI = 1) and measured their expression levels. The results showed that PRRSV infection significantly upregulated the expression of CSF1, CSF2, and CSF3 in MARC-145 cells (Fig. 1C). To study the effects of CSF1, CSF2, and CSF3 on PRRSV infection, MARC-145 cells were transfected with their overexpression plasmids and infected with PRRSV, and the viral replication level was detected. As shown in Fig. 1D and E, overexpression of CSF2 significantly inhibited PRRSV replication. Subsequently, MARC-145 cells were transfected with the CSF2 overexpression plasmid and infected with PRRSV, and the viral replication levels at different time points were detected. The results showed that CSF2 overexpression inhibited PRRSV replication at different time points (Fig. 1F and G). The effect of PRRSV infection on CSF2 expression was further examined. The results showed that PRRSV infection at various time points significantly promoted both CSF2 protein and mRNA expression levels (Fig. 1H and I). These findings indicated that PRRSV infection significantly promoted CSF2 expression, and CSF2 overexpression could significantly inhibit PRRSV replication.

### CSF2 upregulates IL15 to inhibit PRRSV replication

**Fig 2:**
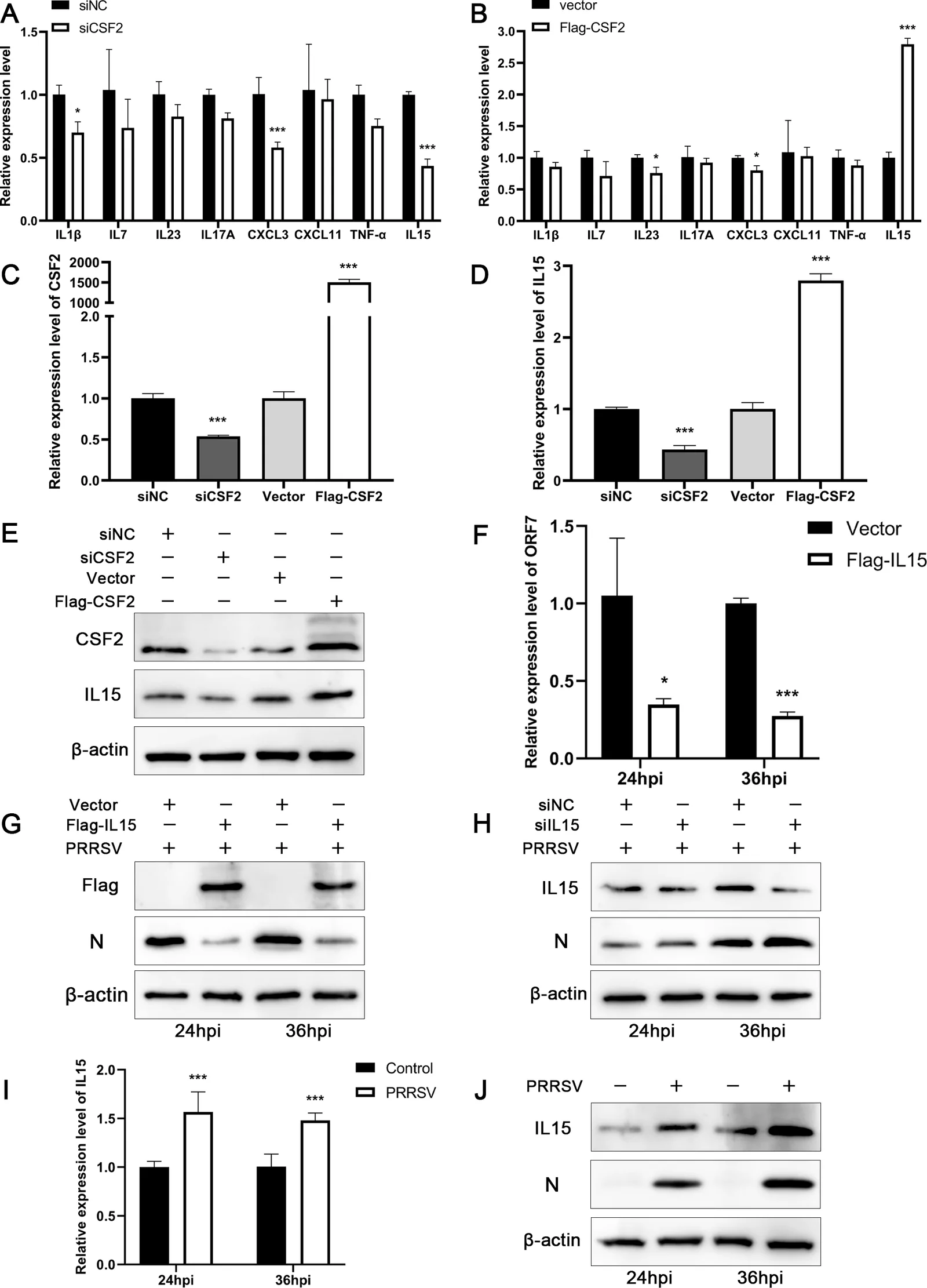
CSF2 upregulates IL15 to inhibit PRRSV replication. (**A and B**) MARC-145 cells were transfected with CSF2 siRNA or CSF2 overexpression plasmid, and the mRNA expression levels of the indicated cytokines were detected at 24 hpi. (**C–E**) MARC-145 cells were transfected with CSF2 siRNA or CSF2 overexpression plasmid, and the protein and mRNA expression levels of CSF2 and IL15 were detected at 24 hpi. (**F–H**) MARC-145 cells were transfected with an IL15 overexpression plasmid or IL15 siRNA, then infected with PRRSV, and PRRSV replication levels were detected at 24 and 36 hpi. (**I and J**) MARC-145 cells were infected with PRRSV, and the protein and mRNA expression levels of IL15 were detected at 24 and 36 hpi. *, *P*  <  0.05; **, *P*  <  0.01; ***, *P*  <  0.001 compared with the respective control.

Studies have reported that CSF2 plays an important role in regulating the expression of cytokines (25). To investigate how CSF2 inhibits PRRSV replication, we transfected MARC-145 cells with CSF2 siRNA or an overexpression plasmid and measured the expression levels of related cytokines. As shown in Fig. 2A and B, knockdown of CSF2 significantly inhibited the expression of IL1β, CXCL3, and IL15, while overexpression of CSF2 significantly promoted the expression of IL15. To further examine the regulatory effect of CSF2 on IL15, MARC-145 cells were transfected with CSF2 siRNA or an overexpression plasmid, and the mRNA and protein expression levels of IL15 were measured. The results showed that CSF2 knockdown significantly suppressed IL15 expression, whereas CSF2 overexpression significantly promoted IL15 expression, indicating that CSF2 positively regulates IL15 expression (Fig. 2C through E). To further investigate the effect of IL15 on PRRSV infection, we transfected MARC-145 cells with the overexpression plasmid or siRNA of IL15 and measured the replication level of PRRSV at different time points. As shown in Fig. 2F through H, overexpression of IL15 significantly inhibits the replication of PRRSV, while knockdown of IL15 significantly promotes the replication of PRRSV. Furthermore, the effect of PRRSV infection on IL15 expression was examined. The results revealed that PRRSV infection significantly upregulated the expression of IL15 (Fig. 2I and J). These results suggested that CSF2 can inhibit PRRSV replication by promoting IL15 expression.

### FRA1 activates CSF2 expression

**Fig 3:**
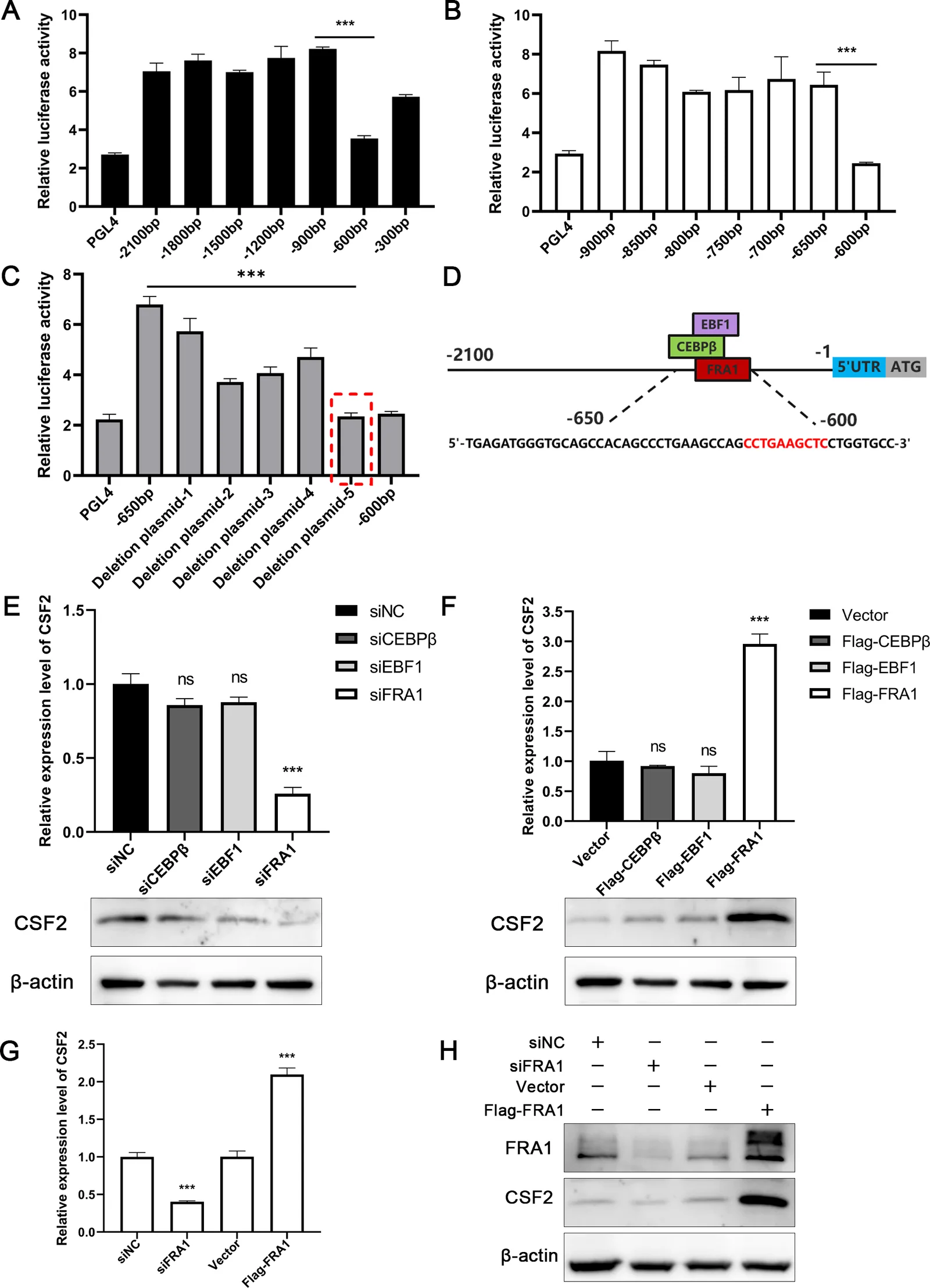
FRA1 activates CSF2 expression. (**A and B**) HEK-293T cells were transfected with CSF2 promoter truncated plasmids, respectively. The dual-luciferase activity of the promoter truncated plasmids was detected 24 h after transfection. (**C and D**) HEK-293T cells were transfected using CSF2 promoter deletion plasmids, respectively. The dual-luciferase activity of the promoter deletion plasmids was detected 24 h after transfection. The corresponding sequences of the deletion plasmids were submitted to PROMO online software to predict the possible binding transcription factors. (**E and F**) MARC-145 cells were transfected with the predicted transcription factor siRNA or overexpression plasmids, and the mRNA and protein expression levels of CSF2 were detected 24 h later. (**G and H**) MARC-145 cells were transfected with siRNA or an overexpression plasmid of FRA1, and the mRNA and protein expression levels of CSF2 were detected 24 h later. *, *P* < 0.05; **, *P* < 0.01; ***, *P* < 0.001 compared to the respective control.

To explore how host cells upregulate CSF2 to exert antiviral functions, we identified the upstream transcription factors of CSF2. First, we constructed the promoter region plasmid of CSF2 (−2,100 bp) and determined its luciferase activity through a dual-luciferase reporter system. Then, the promoter plasmid was gradually truncated, and the luciferase activity was measured. The results showed that the activity in the −600 bp region was significantly reduced, indicating that the region between −900 bp and −600 bp is a potential transcription factor binding site (Fig. 3A). Subsequently, gradient truncation was performed within this area, and the luciferase activity was detected. The results showed that the range of −650 to −600 bp was a potential transcription factor binding site (Fig. 3B). Within this sequence (50 bp), five deletion mutant promoter plasmids were sequentially constructed, and their luciferase activities were measured. The results showed that the fifth deletion promoter plasmid exhibited the lowest activity, indicating that this deletion sequence was a potential transcription factor binding site (Fig. 3C). We submitted this sequence (red) to the PROMO website to predict the transcription factors that might bind to this sequence. As shown in Fig. 3D, the transcription factors predicted to bind this sequence were CEBPβ, EBF1, and FRA1. To further validate the effect of the predicted transcription factors on CSF2 expression, we transfected MARC-145 cells with siRNA or overexpression plasmids targeting CEBPβ, EBF1, and FRA1 and measured CSF2 mRNA and protein levels. The results showed that FRA1 was the most significant transcription factor regulating CSF2 expression. Knockdown of FRA1 significantly suppressed both CSF2 mRNA and protein levels, while its overexpression markedly promoted CSF2 expression (Fig. 3E and F). To further examine the effect of FRA1 on CSF2 expression, we transfected cells with FRA1 siRNA or the overexpression plasmid and measured CSF2 mRNA and protein expression levels. The results showed that FRA1 knockdown significantly inhibited CSF2 expression, while FRA1 overexpression significantly promoted CSF2 expression (Fig. 3G and H), indicating that FRA1 is an upstream transcription factor that activates CSF2 expression.

### FRA1 upregulates the CSF2-IL15 signaling pathway

**Fig 4:**
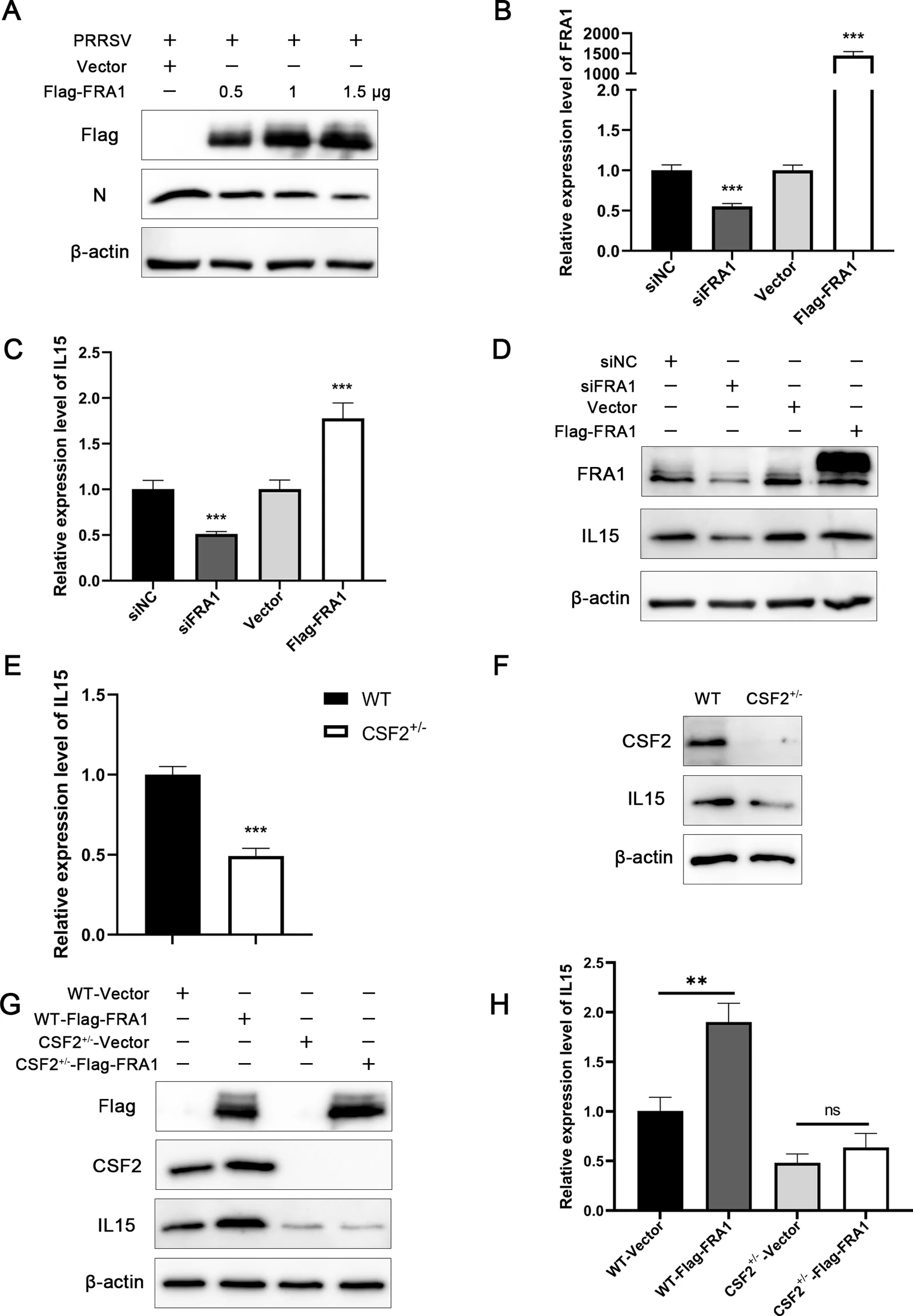
FRA1 upregulates the CSF2-IL15 signaling pathway. (**A**) MARC-145 cells were transfected with different amounts of FRA1 overexpression plasmids; the cells were infected with PRRSV, and the level of virus replication was measured at 24 hpi. (**B–D**) MARC-145 cells were transfected with siRNA or overexpression plasmid of FRA1, and the mRNA and protein expression levels of FRA1 and IL15 were detected 24 h after transfection. (**E and F**) MARC-145 (WT) and MARC-145-CSF2^+/−^ (CSF2^+/−^) cells were transfected with FRA1 overexpression plasmids, and the expression levels of CSF2 and IL15 were detected 24 h after transfection. (**G and H**) MARC-145 and MARC-145-CSF2^+/−^ cells were transfected with the FRA1 overexpression plasmid, and the expression levels of CSF2 and IL15 were detected 24 h after transfection. *, *P* < 0.05; **, *P* < 0.01; ***, *P* < 0.001 compared with the respective control.

We found that FRA1 promotes CSF2 expression; hence, we investigated the effect of FRA1 on PRRSV replication. The results found that overexpression of FRA1 significantly inhibited PRRSV replication (Fig. 4A). To further investigate whether FRA1 exerts its anti-PRRSV effect by promoting the CSF2-IL15 pathway, MARC-145 cells were transfected with FRA1 siRNA or the overexpression plasmid, and IL15 expression was measured. As shown in Fig. 4B through D, knockdown of FRA1 significantly inhibited IL15 expression, while its overexpression significantly promoted IL15 expression. Subsequently, to investigate whether the regulation of IL15 expression by FRA1 depends on CSF2, we first constructed a CSF2 knockout cell line (MARC-145-CSF2^+/−^) to detect the effect of CSF2 knockout on the expression of IL15. The results showed that the expression of IL15 in MARC-145-CSF2^+/−^ cells was significantly lower than that in MARC-145 cells (Fig. 4E and F). We further transfected a FRA1 overexpression plasmid into MARC-145 and MARC-145-CSF2^+/−^ cells and tested the IL15 expression levels. As shown in Fig. 4G and H, overexpression of FRA1 significantly promoted IL15 expression in MARC-145 cells, whereas it did not significantly upregulate IL15 expression in MARC-145-CSF2^+/−^ cells. These results indicated that FRA1-mediated promotion of IL15 expression requires the involvement of CSF2.

### PRRSV induces FRA1 protein degradation

**Fig 5:**
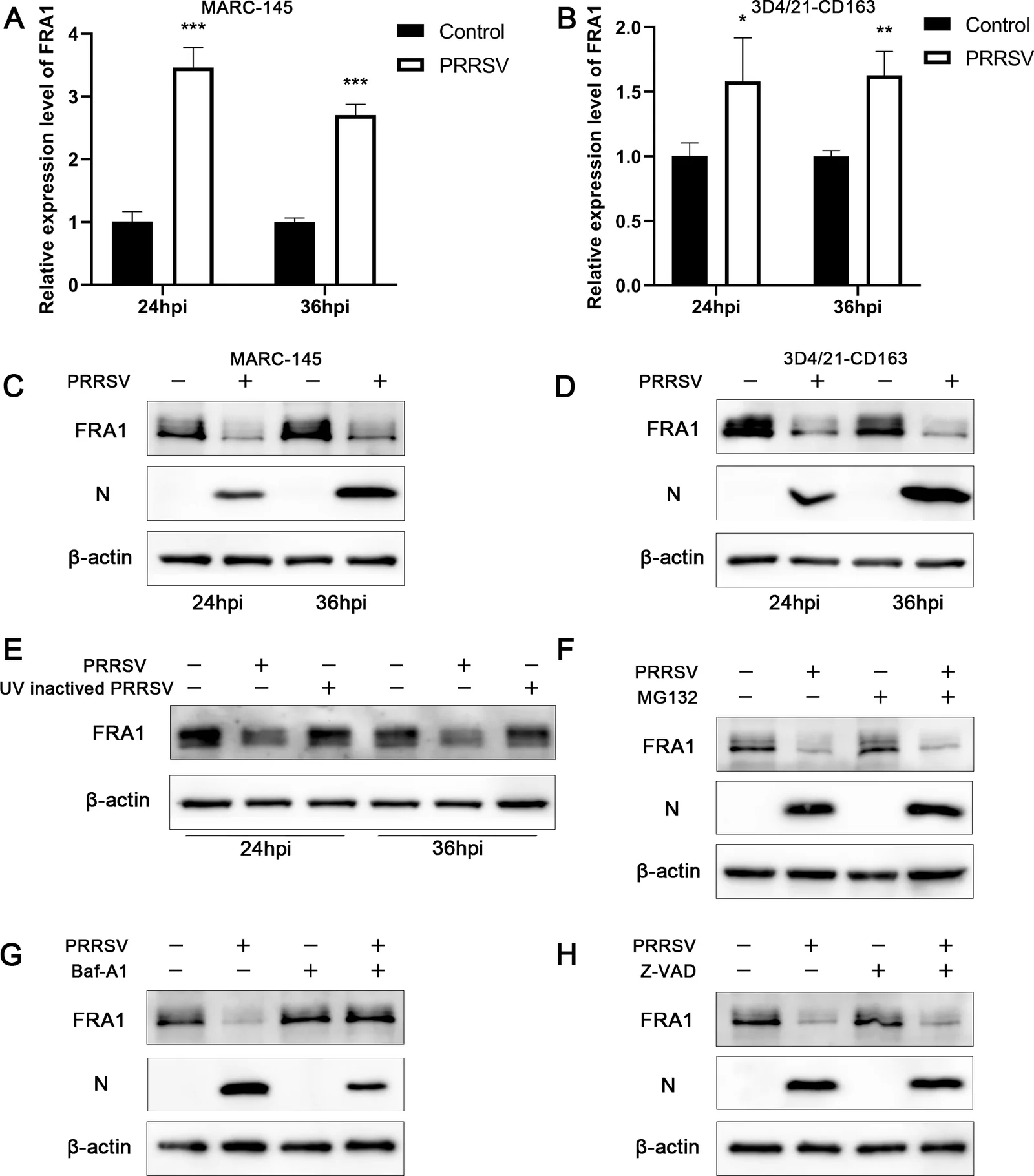
PRRSV induces FRA1 protein degradation. (**A–D**) MARC-145 and 3D4/21-CD163 cells were infected with PRRSV, and the mRNA and protein expression levels of FRA1 were detected at 24 and 36 hpi. (**E**) MARC-145 cells were infected with PRRSV or UV-inactive PRRSV, and the protein expression level of FRA1 was detected at 24 and 36 hpi. (**F–H**) MARC-145 cells infected with or without PRRSV were treated with inhibitors, and FRA1 protein expression levels were measured at 24 hpi. *, *P* < 0.05; **, *P* < 0.01; ***, *P* < 0.001 compared with the respective control.

To investigate the changes in FRA1 expression following PRRSV infection, we infected MARC-145 and 3D4/21-CD163 cells (immortalized PAMs) with PRRSV and measured FRA1 mRNA and protein levels. The results showed that PRRSV infection significantly upregulated FRA1 mRNA levels in MARC-145 and 3D4/21-CD163 cells (Fig. 5A and B). Notably, the protein level of FRA1 was significantly decreased after PRRSV infection (Fig. 5C and D). We therefore hypothesized that PRRSV induces post-translational modifications, leading to the degradation of FRA1 protein, thereby explaining the observed increase in mRNA but decrease in protein. To determine whether PRRSV infection directly causes FRA1 degradation, we infected MARC-145 cells with either UV-inactivated PRRSV or uninactivated virus and assessed FRA1 protein levels. As shown in Fig. 5E, PRRSV infection significantly reduced FRA1 protein levels, whereas UV-inactivated virus did not. To further elucidate the pathway through which PRRSV degrades FRA1, we treated PRRSV-infected cells with the proteasome inhibitor (MG132), autophagy inhibitor (bafilomycin A1 and Baf-A1), and apoptosis inhibitor (Z-VAD), respectively, followed by the detection of FRA1 protein levels. The results revealed that treatment with the autophagy inhibitor Baf-A1 significantly blocked PRRSV-mediated degradation of FRA1 protein, while the proteasome inhibitor MG132 and the apoptosis inhibitor Z-VAD had no significant effect (Fig. 5F through H). This indicates that PRRSV infection can degrade FRA1 via the autophagy-lysosomal pathway.

### PRRSV upregulates HSPA1B expression to degrade FRA1

**Fig 6:**
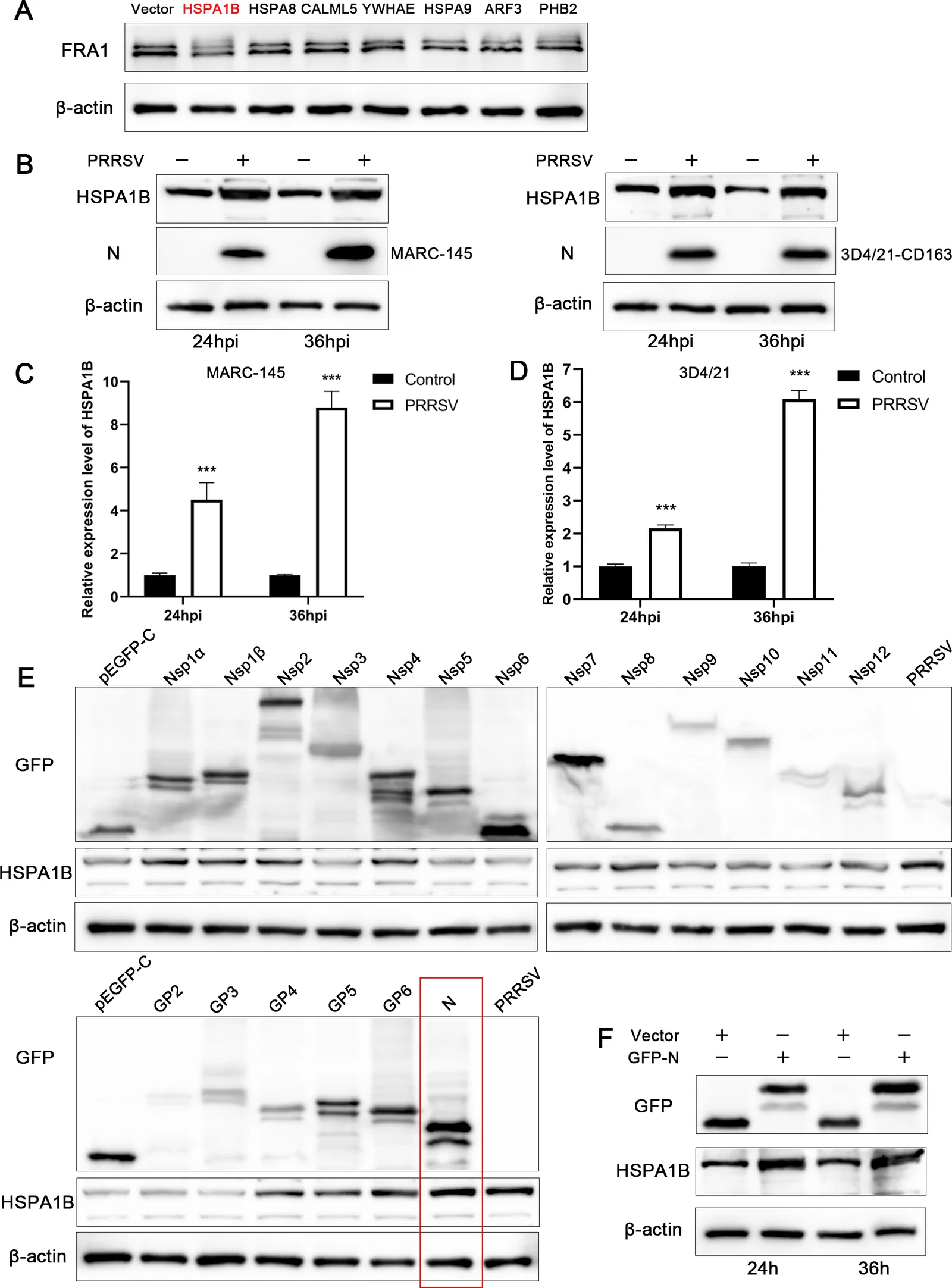
PRRSV upregulates HSPA1B expression to degrade FRA1. (**A**) MARC-145 cells were transfected with a vector or an overexpression plasmid of autophagy-related proteins, respectively, and the protein expression level of FRA1 was detected 24 h after transfection. (**B–D**) MARC-145 or 3D4/21-CD163 cells were infected with PRRSV, and the protein and mRNA expression levels of HSPA1B were detected at 24 and 36 hpi. (**E**) The structural and non-structural protein plasmids of PRRSV were transfected into 3D4/21-CD163 cells, and the protein expression level of HSPA1B was detected 24 h after transfection. (**F**) MARC-145 cells were transfected with the N protein plasmid, and the expression of HSPA1B protein was detected 24 and 36 h after transfection. *, *P* < 0.05; **, *P* < 0.01; ***, *P* < 0.001 compared with the respective control.

To explore how PRRSV degrades FRA1, we transfected HEK-293T cells with a FRA1 overexpression plasmid and then identified proteins interacting with FRA1 using liquid chromatography-tandem mass spectrometry (LC-MS/MS). The results revealed that multiple autophagy-related proteins interact with FRA1 (Table 1). To determine which autophagy-related protein facilitates FRA1 degradation, we constructed overexpression plasmids for the seven proteins with the highest interaction scores and transfected them separately into MARC-145 cells to assess FRA1 protein levels. The results showed that the overexpression of HSPA1B significantly reduced the protein level of FRA1 (Fig. 6A), indicating that HSPA1B is the main autophagy-related protein promoting FRA1 degradation. Studies have reported that HSPA1B, a member of the heat shock protein 70 family, plays a central role in CMA by recognizing and binding target proteins containing the KFERQ motif and mediating their degradation via the lysosomal pathway (26). To investigate the effect of PRRSV infection on HSPA1B-mediated CMA, we first examined the changes in HSPA1B expression following PRRSV infection. As shown in Fig. 6B through D, PRRSV infection significantly upregulated HSPA1B expression in both MARC-145 and 3D4/21-CD163 cells. To identify the specific PRRSV structural component responsible for promoting HSPA1B upregulation, we transfected 3D4/21-CD163 cells with plasmids expressing individual PRRSV non-structural or structural proteins and measured HSPA1B expression. The results demonstrated that the N protein was the most potent viral protein in enhancing HSPA1B expression (Fig. 6E). Further analysis confirmed that the N protein significantly increases HSPA1B expression (Fig. 6F). These results indicate that HSPA1B can significantly promote the degradation of FRA1 protein, and PRRSV N protein is the most prominent viral protein that upregulates HSPA1B expression.

**TABLE 1:** A list of the top 10 proteins interacting with the FRA1 protein

Protein names	Gene names	Number of proteins	Peptides FRA1	Unique peptides FRA1	LFQ intensity FRA1
Heat shock 70 kDa protein 1B	HSPA1B	19	13	5	8,140,800
Heat shock cognate 71 kDa protein	HSPA8	28	8	6	5,853,300
Calmodulin-like protein 5	CALML5	2	2	2	559,950
14-3-3 protein epsilon	YWHAE	11	4	3	545,590
Stress-70 protein, mitochondrial	HSPA9	17	4	4	255,790
Heat shock protein HSP 90-beta	HSP90AB1	8	2	1	230,000
78 kDa glucose-regulated protein	HSPA5	5	4	3	225,550
ADP-ribosylation factor 3	ARF3	18	1	1	112,480
Prohibitin-2	PHB2	12	1	1	74,045
Small ubiquitin-related modifier 2	SUMO2	2	1	1	61,649

### PRRSV N protein interacts with HSPA1B and FRA1

**Fig 7:**
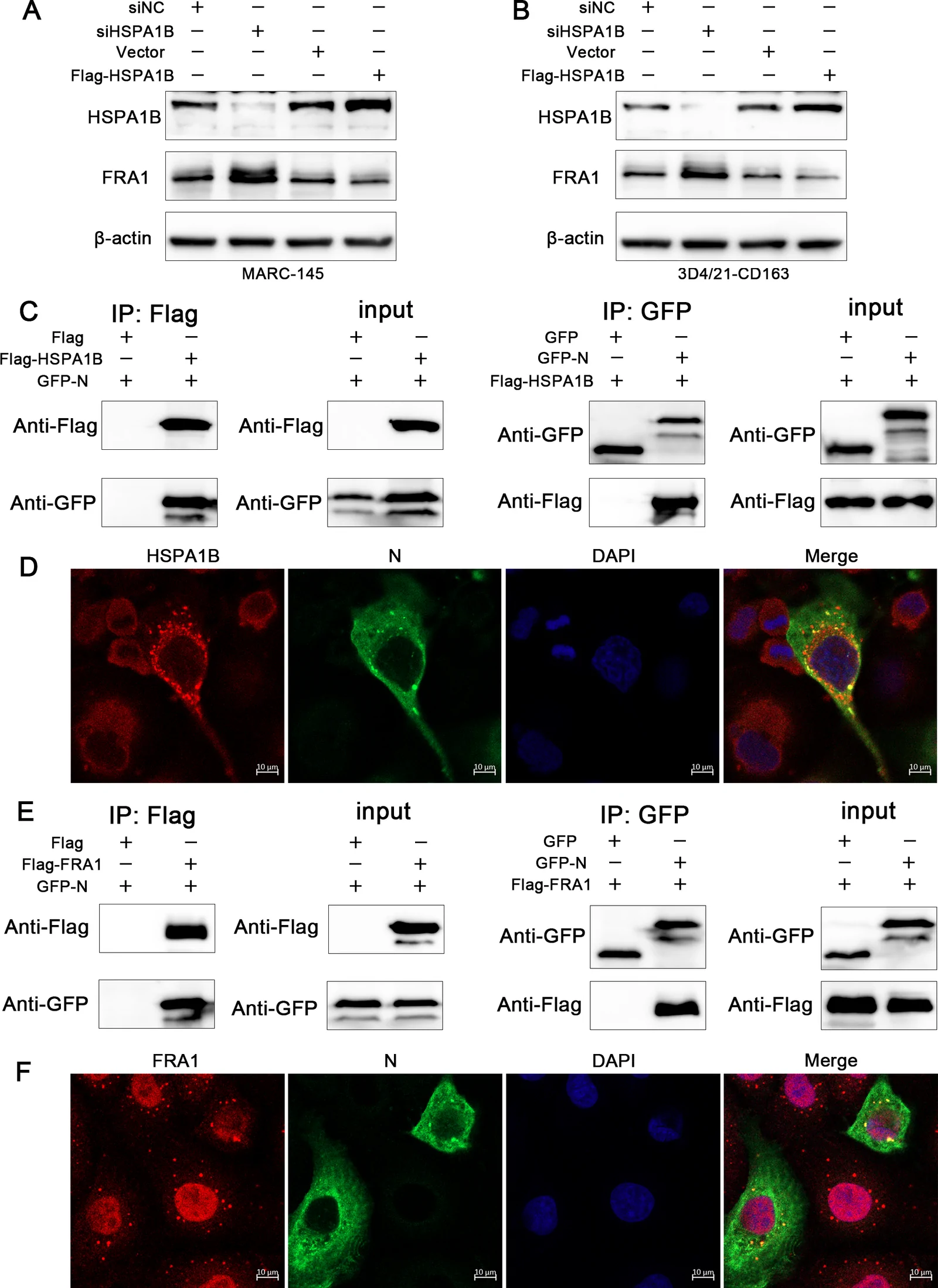
PRRSV N protein interacts with HSPA1B and FRA1. (**A and B**) HSPA1B siRNA or an overexpression plasmid was transfected into MARC-145 and 3D4/21-CD163 cells, and the protein expression levels of HSPA1B and FRA1 were detected 24 h after transfection. (**C and D**) The HSPA1B and N protein plasmids were co-transfected into HEK-293T cells, and coimmunoprecipitation and immunofluorescence detection were performed 24 h after transfection. (**E and F**) The FRA1 and N protein plasmids were co-transfected into HEK-293T cells, and coimmunoprecipitation and immunofluorescence detection were performed 24 h after transfection.

To explore how HSPA1B degraded FRA1, we transfected the siRNA or overexpression plasmid of HSPA1B into MARC-145 and 3D4/21-CD163 cells and detected the protein level of FRA1. The results showed that knockdown of HSPA1B significantly enhanced FRA1 expression, while the overexpression of HSPA1B significantly suppressed it (Fig. 7A and B). To further investigate the interactions between the PRRSV N protein and HSPA1B or FRA1, we employed molecular docking to predict the binding of the N protein with HSPA1B as well as with FRA1. The prediction results showed that the PRRSV N protein can interact with both HSPA1B and FRA1 (Fig. S1A and B). Subsequently, we co-transfected HEK-293T cells with plasmids expressing the N protein and HSPA1B and performed coimmunoprecipitation assays. The results showed that the N protein could interact with HSPA1B (Fig. 7C). The interaction between the N protein and HSPA1B was detected by confocal immunofluorescence microscopy, and consistent results were also observed (Fig. 7D); the fluorescence intensity of HSPA1B was also significantly enhanced in PRRSV-positive cells. We found an interaction between HSPA1B and FRA1 in a previous LC-MS/MS proteomic analysis; hence, we hypothesized that the N protein could also interact with FRA1. We further co-transfected HEK-293T cells with plasmids expressing the N protein and FRA1 and performed a coimmunoprecipitation assay. The results confirmed that the N protein interacts with FRA1 (Fig. 7E). The interaction between the N protein and HSPA1B was further detected by confocal immunofluorescence microscopy, and the interaction between them was also observed (Fig. 7F). The fluorescence intensity of FRA1 was significantly reduced in PRRSV-positive cells.

### PRRSV N protein enhances HSPA1B-mediated FRA1 degradation

**Fig 8:**
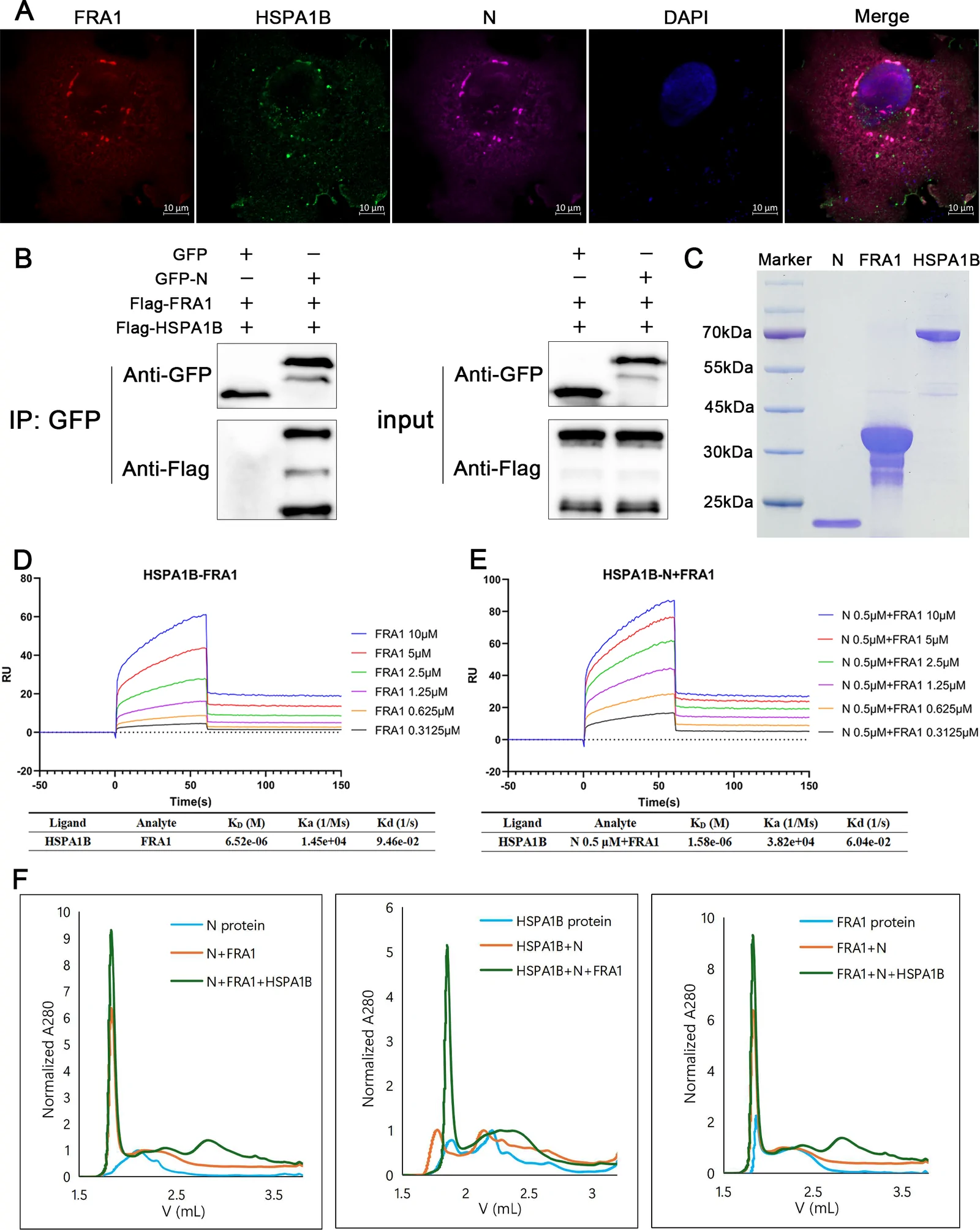
PRRSV N protein enhances HSPA1B-mediated FRA1 degradation. (**A**) The FRA1, HSPA1B, and N protein expression plasmids were co-transfected into MARC-145 cells, and immunofluorescence detection was performed 24 h after transfection. (**B**) The FRA1, HSPA1B, and N protein plasmids were co-transfected into HEK-293T cells, and coimmunoprecipitation detection was performed 24 h after transfection. (**C**) The PRRSV N, FRA1, and HSPA1B proteins were expressed using a prokaryotic expression system, then purified with a nickel column, and their purification results were examined by SDS-PAGE. (**D and E**) The prokaryotically expressed PRRSV N, FRA1, and HSPA1B proteins were used for SPR assays to analyze their binding interactions. (**F**) The prokaryotically expressed PRRSV N, FRA1, and HSPA1B proteins were subjected to SEC analysis, and their elution profiles were examined.

To further investigate the interactions among the PRRSV N protein, HSPA1B, and FRA1, we co-transfected HEK-293T cells with plasmids expressing the N protein, HSPA1B, and FRA1 and examined their interactions. As shown in Fig. 8A and B, immunofluorescence detection revealed triple co-localization of the N protein with HSPA1B and FRA1. Co-immunoprecipitation assays also showed that the PRRSV N protein can simultaneously interact with both HSPA1B and FRA1. To further explore the interactions among the PRRSV N protein, HSPA1B, and FRA1, we performed prokaryotic expression of these three proteins (Fig. 8C) and analyzed their interactions using surface plasmon resonance (SPR) technology. The HSPA1B protein was immobilized on a CM5 sensor chip to evaluate its binding with the FRA1 protein. The SPR results showed that the dissociation constant (K_D_) between HSPA1B and FRA1 was 6.52e–06 M, indicating that HSPA1B can bind to FRA1 with relatively high affinity (Fig. 8D). Subsequently, 0.5 μM of the N protein was introduced into this assay system to test its effect on the affinity and kinetics of the HSPA1B-FRA1 interaction. The results revealed that the presence of the N protein reduced the K_D_ for the HSPA1B-FRA1 binding to 1.58e–06 M, demonstrating that the N protein enhances the binding efficiency between HSPA1B and FRA1 (Fig. 8E). Furthermore, size-exclusion chromatography (SEC) was performed to further verify the formation of a ternary complex among the N protein, HSPA1B, and FRA1. The elution profile showed that the elution volume of the complex was significantly decreased and the corresponding molecular weight was increased after co-incubation of the three proteins, indicating that they can form a stable ternary complex *in vitro* (Fig. 8F). These findings suggest that the PRRSV N protein can promote the binding of HSPA1B to FRA1, thereby enhancing HSPA1B-mediated degradation of FRA1.

### PRRSV N protein-mediated FRA1 degradation requires HSPA1B

**Fig 9:**
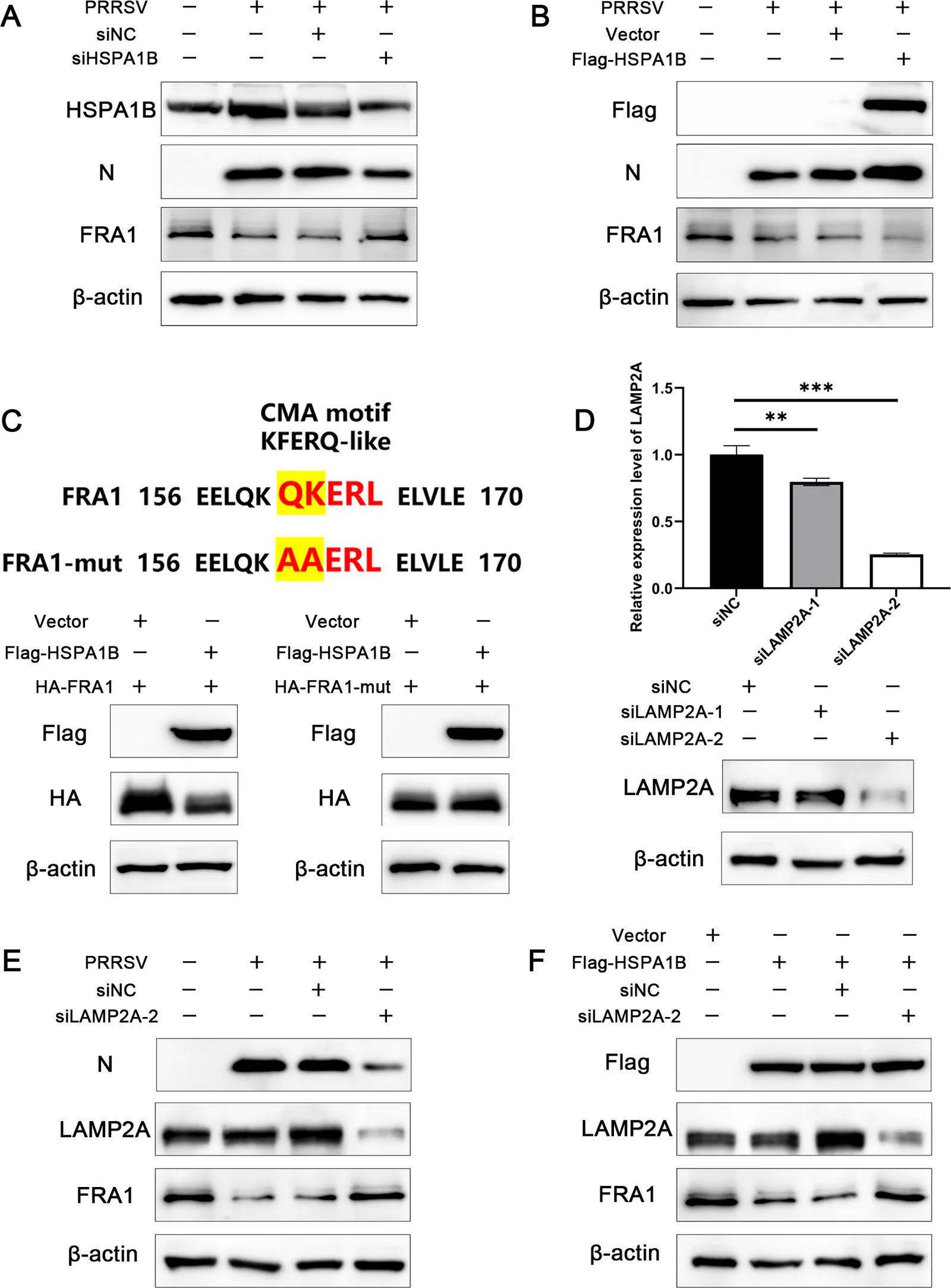
PRRSV N protein-mediated FRA1 degradation requires HSPA1B. (**A and B**) MARC-145 cells were transfected with HSPA1B siRNA or an overexpression plasmid and then infected with PRRSV, and the protein expression levels of HSPA1B and FRA1 were detected at 24 hpi. (**C**) Amino acid mutations were introduced into the KFERQ-like motif within the FRA1 protein sequence. Cells were then co-transfected with either the wild-type FRA1 plasmid or the FRA1-mut plasmid together with the HSPA1B plasmid, and target protein expression was subsequently detected. (**D**) A knockdown sequence targeting LAMP2A was synthesized and transfected into MARC-145 cells, and the mRNA and protein expression levels of LAMP2A were examined. (**E**) MARC-145 cells were transfected with LAMP2A siRNA and then infected with PRRSV, and the expression levels of target proteins were detected. (**F**) MARC-145 cells were transfected with LAMP2A siRNA and an HSPA1B overexpression plasmid, and the expression levels of target proteins were detected. *, *P* < 0.05; **, *P* < 0.01; ***, *P* < 0.001 compared with the respective control.

To further investigate the role of HSPA1B in PRRSV-mediated FRA1 degradation, we infected cells with PRRSV after transfection with or without HSPA1B-targeting siRNA and then examined the protein expression level of FRA1. As shown in Fig. 9A, the expression of FRA1 was significantly reduced after PRRSV infection, while HSPA1B knockdown markedly impaired the PRRSV-mediated degradation of FRA1. Subsequently, we transfected cells with an HSPA1B overexpression plasmid followed by PRRSV infection and detected FRA1 expression. The results showed that PRRSV infection significantly reduced FRA1 expression, and overexpression of HSPA1B further exacerbated this effect (Fig. 9B). These results indicate that PRRSV-mediated degradation of FRA1 requires HSPA1B. To verify whether HSPA1B recognizes the KFERQ-like motif in the FRA1 protein, we mutated this motif in FRA1 and assessed HSPA1B-mediated degradation. As shown in Fig. 9C, mutation of the KFERQ-like motif in FRA1 impeded HSPA1B-induced degradation. Studies have reported that CMA requires the involvement of LAMP2A (26). Therefore, we transfected cells with LAMP2A siRNA and infected them with PRRSV or transfected them with an HSPA1B overexpression plasmid to assess FRA1 protein degradation. The results showed that LAMP2A knockdown hindered FRA1 degradation induced by either PRRSV infection or HSPA1B transfection (Fig. 9D through F). These findings indicate that PRRSV-induced degradation of FRA1 requires LAMP2A involvement. In addition, we further explored the effects of PRRSV N protein and HSPA1B on cellular macroautophagy, and we co-transfected the GFP-LC3 plasmid with the expression plasmid of PRRSV N or HSPA1B into the cells and detected the macroautophagy level of the cells. The results showed that transfection with either PRRSV N or HSPA1B significantly increased the formation of autophagosomes in cells (Fig. S2A). Ectopic expression of PRRSV N or HSPA1B promoted the expression of LC3-II in a dose-dependent manner (Fig. S2B and C). Furthermore, transfection of the N protein expression plasmid promoted HSPA1B expression and inhibited FRA1 expression in a dose-dependent manner (Fig. S2D through G). These results indicate that the PRRSV N protein and HSPA1B can also significantly enhance the level of cellular macroautophagy.

## DISCUSSION

The antagonism of pro-inflammatory cytokine production by PRRSV is a core component of its immune evasion strategy, which directly affects the persistent infection and pathological damage of the virus in the host (27). Studies have shown that PRRSV significantly inhibits the expression of pro-inflammatory cytokines such as IL-1β, IL-6, and TNFα by targeting and regulating key inflammatory signaling pathways such as NF-κB and MAPK (28). For example, the viral nonstructural protein Nsp10 blocks the degradation of IκBα, thereby inhibiting NF-κB nuclear translocation and attenuating transcriptional activation of pro-inflammatory mediators in macrophages (29). Furthermore, PRRSV infection induces the production of anti-inflammatory cytokines (such as IL-10), forming a negative feedback loop that further inhibits the establishment of a pro-inflammatory environment (30). Notably, excessive inhibition of pro-inflammatory responses may exacerbate immune imbalances, allowing the virus to replicate more efficiently in a “low-inflammation” environment (31). Meanwhile, the host’s defense ability against secondary bacterial infections has significantly declined, which explains the phenomenon that PRRSV infections are often accompanied by mixed infections such as *Streptococcus suis* and *Klebsiella pneumoniae* in clinical practice (32–34). Consequently, elucidating the reciprocal antagonism between viral invaders and host pro-inflammatory signaling networks is a new strategy to deal with the immunosuppression caused by PRRSV.

Host cytokines play a central role in recognizing viral infections and initiating antiviral defense responses, involving complex molecular recognition, signal transduction, and effector molecule regulatory networks (35–37). As an important transcription factor of the AP-1 family, we found that FRA1 could directly bind to the transcription element in the promoter region of the CSF2 gene to activate its transcriptional expression, and the upregulation of CSF2 further induced the expression of IL-15. As a pleiotropic cytokine, IL-15 significantly enhances the release of antiviral effector molecules by binding to its receptor, thereby inhibiting the replication of PRRSV. Notably, FRA1 may concurrently coordinate inflammatory responses and immune homeostasis during this process, preventing excessive inflammatory damage and highlighting its dual regulatory function. HSPA1B, a key chaperone protein, plays multifaceted roles in maintaining cellular homeostasis by participating in CMA (38). CMA precisely maintains the proteostatic balance by selectively degrading abnormal or redundant proteins with the KFERQ motif (39, 40). It forms a synergistic network with the ubiquitin-proteasome system and other autophagy pathways (41). During viral infection, CMA directly exerts antiviral or pro-viral infection effects by degrading viral components or inhibiting host factors that are resistant to viral replication (22).

However, PRRSV has evolved multiple immune evasion strategies to counteract host defenses (42). Studies have reported that PRRSV nonstructural protein Nsp2 can block interferon pathway activation by inhibiting SH3KBP1 phosphorylation or hijacking the host ubiquitination system to degrade key signaling molecules (4). This study reveals for the first time that PRRSV N protein hijacks the host heat shock protein HSPA1B to degrade FRA1 through the CMA pathway, thereby antagonizing the CSF2-IL-15 signaling axis. This discovery proposes a novel “virus-autophagy degradation-immune evasion” regulatory mechanism. Our findings demonstrate that while host cells establish multi-layered defense networks through cytokine signaling upon viral invasion, PRRSV counteracts these immune responses by exploiting host protein degradation mechanisms to eliminate critical antiviral molecules, ultimately achieving efficient viral replication. This discovery has deciphered the confrontation mechanism of the arms race between PRRSV and hosts, providing a theoretical basis for the development of broad-spectrum antiviral therapies targeting host cytokines.

**Fig 10:**
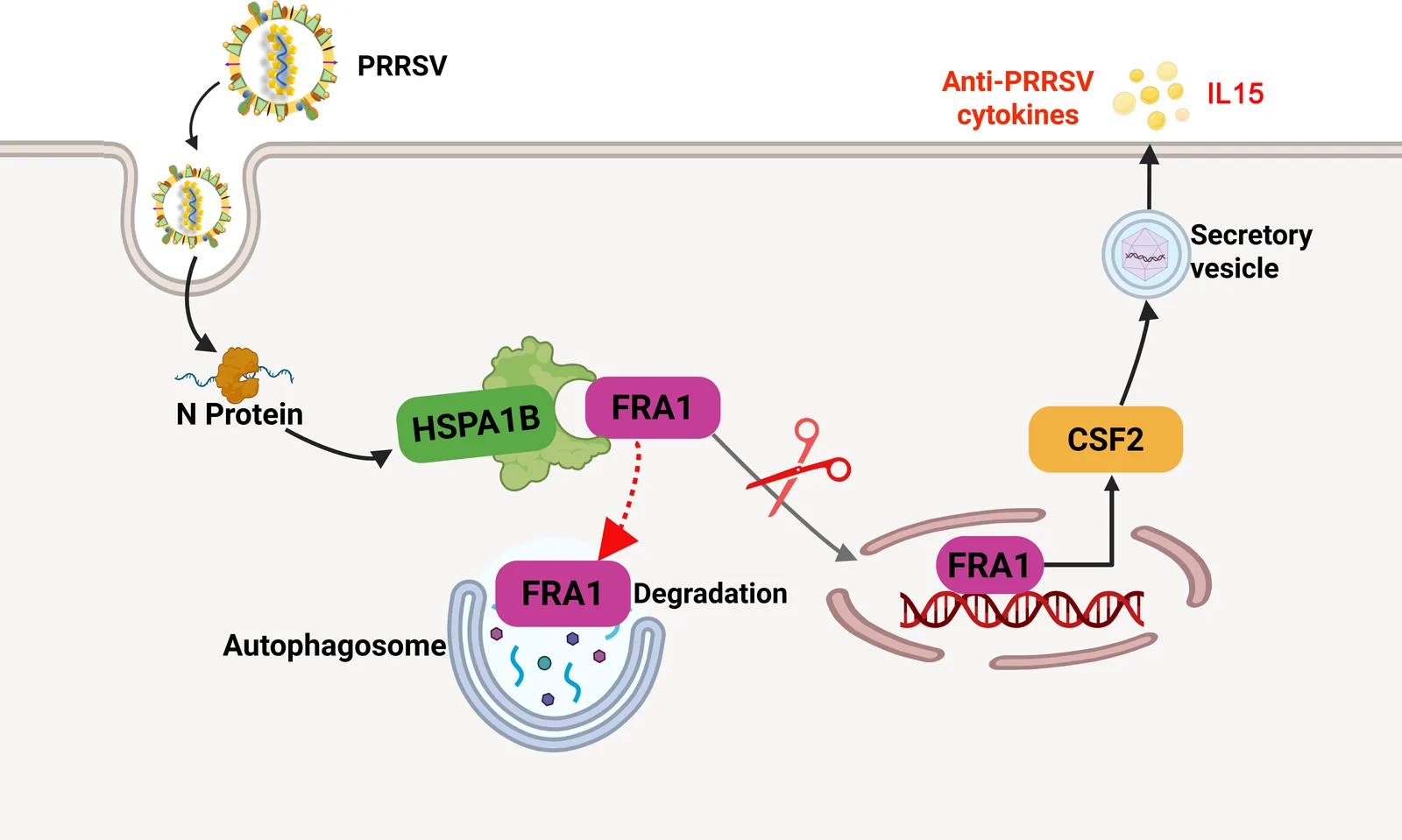
Schematic model of PRRSV antagonizing host antiviral immune response. The PRRSV N protein promotes autophagic degradation of FRA1 by upregulating HSPA1B expression, thereby antagonizing the host antiviral immune responses. The schematic was created with Biorender.com.

In conclusion, we have deciphered a previously unrecognized link between PRRSV immune evasion and chaperone-mediated autophagy. The PRRSV N protein promotes HSPA1B-mediated autophagic degradation of FRA1 to inhibit the CSF2-IL-15 cytokine pathway, thereby enhancing its own replication (Fig. 10). This research not only provides novel insights into the immunosuppressive mechanisms of PRRSV but also offers valuable references for prevention and control of PRRS. This discovery of the PRRSV-host antagonism network unveils actionable targets for combating PRRSV infection.

## MATERIALS AND METHODS

### Cells and virus

PAMs were isolated from 5-week-old PRRSV-negative piglets and cultured in RPMI 1640 medium containing 10% fetal bovine serum. The immortalized porcine alveolar macrophages (3D4/21-CD163), MARC-145, and HEK-293T cells were maintained in DMEM containing 10% fetal bovine serum. The PRRSV GD-HD (GenBank: KP793736.1) strain used in this study was isolated and preserved in our laboratory.

### Antibodies and reagents

Anti-PRRSV N monoclonal antibody was prepared and stored in our laboratory. Anti-β-actin, anti-HA, and anti-GFP monoclonal antibodies were obtained from TransGen Biotech. The BCA kit (PK10026), anti-P62 (18420-1-AP), anti-rabbit IgG antibody labeled with Alexa Fluor 488, and anti-mouse IgG antibody labeled with Alexa Fluor 594 were obtained from Proteintech. Anti-Flag monoclonal antibody was obtained from Beyotime Biotech, and anti-CSF2 (10015-RP02) antibody was obtained from Sino Biological. Anti-LC3 (CY5992) monoclonal antibody was obtained from Abways Biotech. Anti-HSP70 (A12948), anti-ATG4B (A5059), and anti-FRA1 (A26196) monoclonal antibodies were obtained from ABclonal Technology. The Lipofectamine RNAiMAX Transfection Reagent and Lipofectamine 3000 Transfection Reagent were obtained from Thermo Fisher Scientific. siRNA was synthesized by Sangon Biotech.

### RNA-seq analysis

PAMs were seeded in 60-mm culture dishes at a density of 1 × 10⁶ cells per dish and infected with PRRSV GD-HD (MOI = 1). At different time points post-infection, the cells were collected and lysed with TRIzol reagent, and the samples were then sent to a company for RNA-seq analysis. Transcriptome sequencing was performed by Tsingke Biotechnology Co., Ltd. (Beijing, China), and bioinformatic analysis was conducted using the OmicStudio tools.

### RT-qPCR

Total RNA was extracted from cells or culture supernatant with TRIzol, and the cDNA was obtained by reverse transcription with Primescript RT kit (TaKaRa). RT-qPCR analysis was performed using ChamQ SYBR qPCR Master Mix (Vazyme), and the relative expression levels were calculated by the 2^−ΔΔCT^ method. Primers are shown in Table 2.

**TABLE 2:** List of primers used in the RT-qPCR analysis

Primers	Sequence (5′→3′)	Species
gmIL1β-F	CAGATGAAGTGCTCCTTCCAG	Green monkey
gmIL1β-R	TTCTCCATGGCTACAACAACC	
gmIL7-F	GTGAACCCCAACCAACAAAG	Green monkey
gmIL7-R	TGGGAAGTCCAGGAGCATAG	
gmIL23-F	GCCTTCTCTGCTCCCTGATA	Green monkey
gmIL23-R	CTGCTGAGTCTCCCAGTGGT	
gmIL17A-F	ACCGGCAAGGTTAAAGGAAG	Green monkey
gmIL17A-R	AGGGCTTTCTTGCTGCATAA	
gmCXCL3-F	CACACTCAAGAATGGGCAGA	Green monkey
gmCXCL3-R	TGTTCAGCATCTTTCCGATG	
gmCXCL11-F	GAGTGTGAAGGGCATGGCTA	Green monkey
gmCXCL11-R	CCAGGGCCTATGCAAAGACA	
gmTNF-α-F	AACCTCCTCTCTGCCATCAA	Green monkey
gmTNF-α-R	AGTCGAGATAGTCGGGCAGA	
PRRSV-ORF7-F	AATGGCCAGCCAGTCAATCA	PRRSV
PRRSV-ORF7-R	TCATGCTGAGGGTGATGCTG	
gmGAPDH-F	TGGAAAAACCTGCCAAGTACG	Green monkey
gmGAPDH-R	ATGAGGTCCACCACCCTGTT	
gmFRA1-F	CAGTGGATGGTACAGCCTCAT	Green monkey
gmFRA1-R	GAAGTCGGTCAGTTCCTTCCT	
gmIL15-F	GAAGCCAACTGGGTGAATGT	Green monkey
gmIL15-R	TGCTGTTACCTTGCAACTGG	
gmHSPA1B-F	GACTTTGACAACGGAGCTGAG	Green monkey
gmHSPA1B-R	GCATGTGCTCTGTGCTAATGA	
gmCSF1-F	AGACCTCGTGCCAAATTACCT	Green monkey
gmCSF1-R	CTTGACCTTCTCCAGCAACTG	
gmCSF2-F	ATGTGAATGCCATCCAGGAG	Green monkey
gmCSF2-R	AGGGCAGTGCTGCTTGTAGT	
gmCSF3-F	CTGCTTGAGCCAACTCCATAG	Green monkey
gmCSF3-R	CATCTGCTGCCAGATAGTGGT	
pFRA1-F	GACTTCCTGCAGTCGGAGAC	Pig
pFRA1-R	CTTCTGCTTCTGCAGCTCCT	
pHSPA1B-F	AAGATCACCATCACCAACGAC	Pig
pHSPA1B-R	TGTGCTCAAACTCGTCCTTCT	
pGAPDH-F	TGACAACTCCCTCAAGATCG	Pig
pGAPDH-F	AAGCAGGGATGATGTTCTGG	

### Western blot and Co-IP

Following the previously described western blot procedure with minor modifications, the cells were collected and lysed on ice for 25 min. The lysate was centrifuged at 13,000 × *g* for 5 min at 4°C to collect the supernatant. Protein concentration was quantified using a BCA kit. Samples were mixed with loading buffer and boiled for 10 min. Proteins were separated using 12% SDS-PAGE gels and subsequently transferred to PVDF membranes. The membranes were blocked with 5% skim milk in PBST at room temperature for 1 h, followed by incubation with primary antibodies at 4°C overnight. After washing with PBST, the membranes were incubated with secondary antibodies at room temperature for 1 h. Following three additional PBST washes, protein bands were detected using a chemiluminescence detection system.

Magnetic beads were pretreated with PBST. The supernatant of lysed cells was added to anti-HA or anti-Flag magnetic beads. After incubation at room temperature for 3 h, the beads were washed five times with PBST. Loading buffer was added to the beads, and the samples were boiled in water for 10 min before being subjected to western blot analysis.

### Immunofluorescence assay

Cells were fixed with 4% paraformaldehyde at room temperature for 20 min, permeabilized with 0.5% Triton X-100 for 10 min, and then blocked with 3% BSA at room temperature for 1 h. After washing with PBS, the cells were incubated with primary antibody at room temperature for 3 h. Following PBS washes, the cells were incubated with fluorescence-conjugated secondary antibody at room temperature for 1 h. After washing with PBS, the cells were stained with DAPI for 7 min. Finally, after additional PBS washes, fluorescence was captured using a Zeiss fluorescence microscope.

### siRNA and plasmid transfection

The overexpression plasmids and empty vector were transfected with Lipofectamine 3000 Transfection Reagent. siRNA was transfected with Lipofectamine RNAiMAX Transfection Reagent. Cell samples were collected at different treatment time points for testing.

### Dual-luciferase reporter assay

The assay was performed according to a previously described method, with minor modifications (37). MARC-145 cells were seeded into 24-well plates at 80% confluence to assess CSF2 promoter activity. The pGL4-CSF2 promoter plasmid and the pRL-TK control plasmid were co-transfected at a ratio of 100:1. Cells were harvested 36 h post-transfection, and the relative luciferase activity was measured using the Promega dual-luciferase reporter assay kit.

### Protein expression and purification

The gene sequences of PRRSV N protein (GenBank: KP793736.1), FRA1 (XM_007980562.3), and HSPA1B (XM_035295283.3) were synthesized and cloned into the pET28a vector by Tsingke Biotechnology Co., Ltd. The expression plasmids were transformed into *E. coli* strain BL21 (DE3) competent cells by the heat shock method. Protein expression was induced with isopropyl-β-D-thiogalactopyranoside (IPTG). After culture, the culture supernatant and bacterial pellets were harvested separately. Inclusion bodies containing target proteins were solubilized in 8 M urea, and the proteins were subsequently purified using Ni-NTA affinity chromatography columns. The purified proteins were refolded through gradient dialysis and concentrated. Their concentrations were quantified with a BCA protein assay kit for further experiments.

### Statistical analysis

The data were obtained from three replicate experiments and statistically analyzed using GraphPad Prism 9 software, and the results are expressed as the means ± standard deviations (SDs). The significance of differences was analyzed via Student’s *t*-test or one-way analysis of variance (ANOVA), and *P* values of <0.05 (*), <0.01 (**), and <0.001 (***) were considered statistically significant at different levels. *ns* indicates no significant difference.

## Data Availability

Transcriptomics data have been deposited in the Sequence Read Archive (SRA) of the NCBI database, under BioProject accession number PRJNA1309714.

## SUPPLEMENTAL MATERIAL

Supplemental Figures mbio.01091-26-s0001.docx Figures S1 and 2.

ASM does not own the copyrights to Supplemental Material that may be linked to, or accessed through, an article. The authors have granted ASM a non-exclusive, world-wide license to publish the Supplemental Material files. Please contact the corresponding author directly for reuse.
