# Supplementary material for: PRRSV N protein antagonizes host antiviral immune responses by upregulating HSPA1B to induce autophagic degradation of Fos-related antigen 1
Source: mBio. 2026 Aug 21;17(9):e01091-26. doi: 10.1128/mbio.01091-26 (PMC13556196; doi:10.1128/mbio.01091-26)
Supplement: Supplementary file 1 [file mbio.01091-26-s0001.docx]

**Supplementary Figure 1. PRRSV N protein interacts with both HSPA1B and FRA1**

(A-B) Predict the binding interactions of N protein with HSPA1B or FRA1 using molecular docking.

**Supplementary Figure 2. PRRSV N protein and HSPA1B enhance cellular macroautophagy**

(A) MARC-145 cells were co-transfected with PRRSV N or HSPA1B plasmids together with the GFP-LC3 plasmid, respectively, and immunofluorescence assay was performed 24 h after transfection. (B-C) MARC-145 cells were transfected with different amounts of the PRRSV N or HSPA1B plasmid, and western blot analysis was performed 24 h after transfection. (D-F) MARC-145 cells were transfected with different amounts of the PRRSV N protein expression plasmid, and the expression of HSPA1B and FRA1 was detected 24 h post-transfection. (G) MARC-145 cells were transfected with the PRRSV N protein expression plasmid, and FRA1 expression was detected at 24 and 36 h post-transfection.
